# Failure to Rescue After Surgery for Pancreatic Cancer: A Systematic Review and Narrative Synthesis of Risk Factors and Safety Strategies

**DOI:** 10.3390/cancers17193259

**Published:** 2025-10-08

**Authors:** Masashi Uramatsu, Yoshikazu Fujisawa, Paul Barach, Hiroaki Osakabe, Moe Matsumoto, Yuichi Nagakawa

**Affiliations:** 1 Department of Quality and Patient Safety, Tokyo Medical University, Tokyo 160-0023, Japan; fujisaway@myu.ac.jp; 2 Section of Medical Safety Management, Tokyo Medical University Hospital, Tokyo 160-0023, Japan; 3 School of Project Design, Miyagi University, Taiwa 981-3298, Japan; 4 Jefferson College of Population Health, Philadelphia, PA 19107, USA; paul.barach@jefferson.edu; 5 Sheps Health Services Research Center, University of North Carolina, Chapel Hill, NC 27599, USA; 6 Department of Surgery, Imperial College, London SW7 2BX, UK; 7 Department of Gastrointestinal and Pediatric Surgery, Tokyo Medical University, Tokyo 160-0023, Japan; osakabeh@tokyo-med.ac.jp (H.O.); mamt32@tokyo-med.ac.jp (M.M.)

**Keywords:** failure to rescue, pancreatic cancer surgery, postoperative complications, mortality, centralization, perioperative care, risk stratification, non-technical skills, patient safety, quality indicators

## Abstract

**Simple Summary:**

Failure to rescue (FTR)—death after major postoperative complications—is a persistent, variable problem in pancreatic cancer surgery. The review using PRISMA 2020 checklist and flow diagram screened 83 studies (1992–2025) and included 52 studies (2010–2025) across registry, multicenter, single-center, and audit designs. Due to heterogeneity in designs and FTR definitions (in-hospital, 30/90-day, severity- and complication-specific cases), a narrative synthesis was used; no formal risk-of-bias assessment or meta-analysis was performed. FTR varied by definition: pooled rates were 13.2% for 90-day CD ≥ III (G1); 10.3% for in-hospital/30-day CD ≥ III (G3); and 7.4% for 30-day “serious/major” morbidity (G8), with G1 > G3 (+3.0 pp; RR 1.29) and G3 > G8 (+2.9 pp; RR 1.39; all *p* < 0.001). Five domains were consistently linked to lower FTR and improved outcomes: (i) centralization to high-volume centers; (ii) evolution of surgical techniques; (iii) optimized perioperative management (early imaging, structured escalation); (iv) patient-specific risk stratification and prehabilitation; and (v) non-technical skills (NTS) including decision-making, situational awareness, communication, teamwork, and leadership. NTS measurement was infrequent, and no study assessed stress or fatigue management. Future work should standardize pancreas-specific FTR definitions, incorporate process-level rescue metrics, and embed NTS assessments with simulation and implementation science to change and strengthen team behaviors and reduce preventable patient mortality.

**Abstract:**

**Background**: Failure to rescue (FTR), defined as death after major postoperative complications, is a critical quality indicator in pancreatic cancer surgery. Despite advances in surgical techniques and perioperative care, FTR rates remain high and vary across institutions. **Methods**: This systematic review uses a narrative synthesis followed by PRISMA 2020. A PubMed search (1992–2025) identified 83 studies; after screening, 52 studies (2010–2025) were included. Eligible designs were registry-based, multicenter, single-center, or prospective audits. Given substantial heterogeneity in study designs, FTR definitions, and outcome measures, a narrative synthesis was performed; no formal risk-of-bias assessment or meta-analysis was conducted. **Results**: Definitions of FTR varied (in-hospital, 30-day, 90-day, severity-based, and complication-specific cases). Reported rates differed by definition: average reported rates were 13.2% for 90-day CD ≥ III (G1); 10.3% for in-hospital/30-day CD ≥ III (G3); and 7.4% for 30-day “serious/major” morbidity (G8). Absolute differences were +3.0 and +2.9 percentage points (exploratory, descriptive comparisons). Five domains were consistently associated with lower FTR: (i) centralization to high-volume centers; (ii) safe adoption/refinement of surgical techniques; (iii) optimized perioperative management including early imaging and structured escalation pathways; (iv) patient-level risk stratification and prehabilitation; and (v) non-technical skills (NTSs) such as decision-making, situational awareness, communication, teamwork, and leadership. Among NTS domains, stress and fatigue management were not addressed in any included study. **Limitations**: Evidence is predominantly observational with substantial heterogeneity in study designs and FTR definitions; the search was limited to PubMed; and no formal risk-of-bias, publication-bias assessment, or meta-analysis was performed. Consequently, estimates and associations are descriptive/associative with limited certainty and generalizability. **Conclusions**: NTSs were rarely used or measured across the included studies, with validated instruments; quantitative assessment was uncommon, and no study evaluated stress or fatigue management. Reducing the FTR after pancreatic surgery will require standardized, pancreas-specific definitions of FTR, process-level rescue metrics, and deliberate strengthening of NTS. We recommend a pancreas-specific operational definition with an explicit numerator/denominator: numerator = all-cause mortality within 90 days of surgery; denominator = patients who experience major complications (Clavien–Dindo grade III–V, often labeled “CD ≥ 3”). Addressing the gaps in stress and fatigue management and embedding behavioral metrics into quality improvement programs are critical next steps to reduce preventable mortality after complex pancreatic cancer procedures.

## 1. Introduction

Pancreatic resection remains one of the most complex and high-risk surgical procedures, with postoperative morbidity and mortality rates regularly reported as high as 30–70% and 2–15%, respectively [1,2,3,4,5,6]. The pancreatoduodenectomy (PD)—most commonly performed for pancreatic cancer but also for other periampullary diseases—is the most technically demanding, frequently complicated by intra-abdominal fluid collections, postoperative pancreatic fistula (POPF), and postpancreatectomy hemorrhage (PPH) [7,8,9,10,11,12]. While perioperative mortality, particularly in pancreatic cancer surgery, has declined over the past few decades due to advances in surgical techniques, patient selection, and perioperative care [13,14], morbidity remains stubbornly high, and complications such as POPF and PPH continue to pose life-threatening risks and long-term morbidity. Postoperative chemotherapy, crucial for improving long-term survival in pancreatic cancer, can be delayed by poorly managed complications; ineffective early management can undermine optimal outcomes. PPH, often triggered by enzymatic erosion of major vessels, is one of the most fatal events after PD and has been identified as a major cause of interhospital transfers and preventable mortality [15,16,17,18].

Because the rescue pathways in pancreatic surgery are uniquely shaped by POPF and PPH, we restricted our scope to pancreatic procedures to ensure clinical comparability across studies. Increasing attention has been paid to the concept of failure to rescue (FTR)—defined as death following a major postoperative complication—which captures individual, team, and institutional variations in their ability to recognize, intervene, and manage surgical complications before they become fatal [19,20,21,22]. A growing body of research in North America and Europe has examined hospital- and patient-level determinants of FTR [8,23,24,25,26], and a number of promising strategies—such as early postoperative imaging [27,28,29], preoperative frailty screening [30,31], robotic surgery [32,33,34], and timely interprofessional interventions—have been proposed to mitigate FTR in high-risk pancreatic surgery. However, the evidence regarding their effectiveness remains mixed, highlighting important organizational, implementation and cultural challenges.

The PRISMA 2020 checklist was used to guide a systematic review with narrative synthesis examining how FTR is defined and reported after pancreatic surgery and identifying modifiable clinical, organizational, and team-based strategies to reduce FTR. Our research questions were: for adults undergoing pancreatic surgery, (1) what definitions and rates of FTR have been reported, and (2) which modifiable clinical, organizational, patient-level, and team (non-technical) strategies are associated with lower FTR?

## 2. Methods

We conducted a systematic review with a narrative synthesis of existing literature on FTR in pancreatic surgery care, using the PRISMA 2020 guidelines [35]. The review comprised five phases: (1) Identifying the research question, (2) identifying relevant studies, (3) study selection, (4) collating data, and (5) synthesizing results narratively.

Registration statement: The review protocol was not registered in a database such as PROSPERO.

Checklist: A completed PRISMA 2020 checklist is provided in the Supplementary Materials.

PRISMA statement: Reporting follows the PRISMA 2020 guidelines.

### 2.1. Search Strategy

We conducted a systematic literature search in PubMed to identify studies on FTR after pancreatic surgery. Given the distinctive pathophysiology and rescue pathways in pancreatic surgery—particularly the central role of clinically relevant POPF and delayed post-pancreatectomy hemorrhage—we restricted the scope to pancreatic procedures to enhance comparability, ensure clinically coherent rescue determinants, and avoid cross-procedure ecological bias.

The following search query was used:

(“failure to rescue” [All Fields] OR “FTR” [All Fields] OR “failure-to-rescue” [All Fields])

AND (“pancreatic surgery” [All Fields] OR “pancreatectomy” [All Fields] OR “pancreatoduodenectomy” [All Fields] OR “whipple procedure” [All Fields] OR “distal pancreatectomy” [All Fields] OR “total pancreatectomy” [All Fields]) AND (“1 January 1992” [Date-Publication]: “9 May 2025” [Date-Publication])

### 2.2. Selection Criteria

Studies were included in the review (1) if they specifically focused on failure to rescue (FTR) in the context of pancreatic surgery, (2) discussed clinical, institutional, or system-level strategies for reducing FTR and (3) reported relevant outcome measures such as morbidity, mortality, or rescue rates. Eligible study designs included randomized controlled trials, observational studies, and multicenter analyses. Reviews were retrieved at the search stage but excluded during eligibility assessment and were used only as background references. To minimize cross-procedure heterogeneity, the scope was restricted a priori to pancreatic procedures.

Studies were excluded if they (1) were not related to pancreatic surgery (e.g., such as those focusing solely on gastric or colorectal surgery), (2) lacked clinical data (e.g., commentaries or expert opinions), (3) were review articles, or (4) were case reports with insufficient information for meaningful analysis. Studies involving a mixed surgical population were included only when pancreatic surgery patients were clearly represented or analyzable. Studies covering hepato-biliary-pancreatic (HBP) procedures as a combined category were included if pancreatic surgery constituted a substantial and integral component of the analysis. All titles, abstracts, and full texts were independently reviewed by two authors (MU and YN) to determine eligibility. Any disagreements were resolved through discussion until a consensus was reached.

### 2.3. Data Extraction and Synthesis

#### 2.3.1. Data Extraction

We developed a standardized extraction form (available upon request) to capture the study characteristics, patient populations, surgical procedures, definitions of FTR, intervention details (where applicable), and FTR outcome measures. Two reviewers (MU and YN) independently extracted data, and discrepancies were resolved by discussion and consensus.

#### 2.3.2. Definition Framework and Grouping

The cohorts were grouped along three prespecified dimensions to isolate the impact of definitional choices on reported FTR: (i) observation window (in-hospital, 30-day, or 90-day cases); (ii) severity threshold for the qualifying postoperative complication (e.g., Clavien–Dindo ≥ III/IIIa; NSQIP “serious/major”; administrative/registry “any/major”); and (iii) data source (clinical cohort/registry vs. administrative/claims). These dimensions were used to classify each cohort prior to analysis.

#### 2.3.3. Data Synthesis

For each definition grouping, we aggregated deaths and denominators across non-overlapping cohorts and calculated simple pooled proportions with Wilson 95% confidence intervals. To illustrate the magnitude of definition-driven differences, we report unweighted, exploratory contrasts. These calculations are descriptive and hypothesis-generating only and do not constitute a formal meta-analysis; no study-level weighting, heterogeneity modeling, or risk-of-bias assessment was performed. We did not conduct a formal risk-of-bias appraisal nor a formal meta-analysis, owing to substantial heterogeneity across study designs, patient populations, FTR definitions, and outcome measures.

## 3. Results

### 3.1. Characteristics of the Included Studies

**Figure 1:**
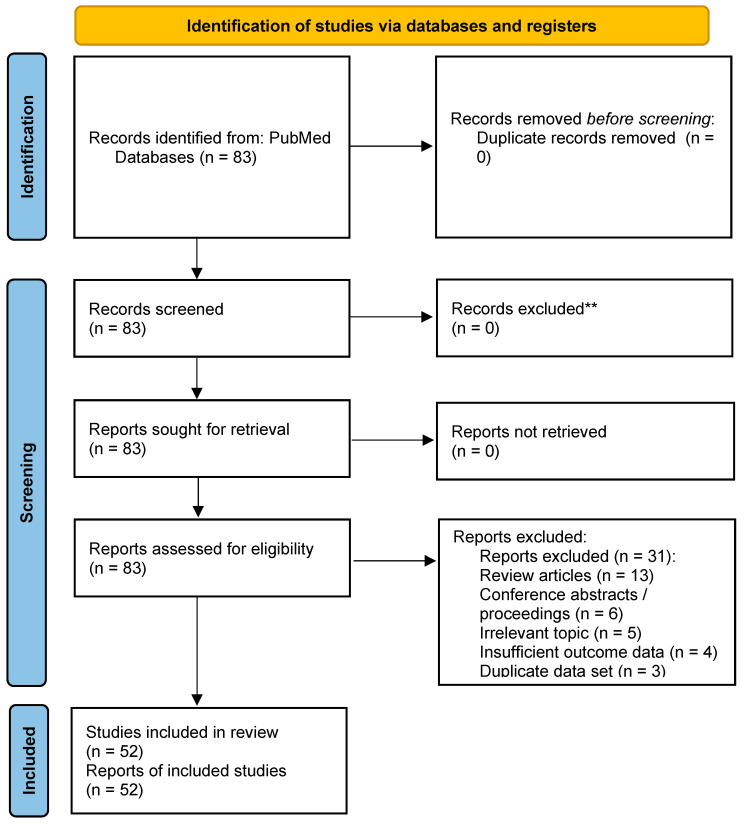
PRISMA 2020 flow diagram of study selection for this systematic review with narrative synthesis on failure to rescue (FTR) after pancreatic surgery. Studies were sequentially excluded if they were unrelated to hepato-biliary-pancreatic surgery, lacked specific reference to FTR, or did not include data on pancreatic surgery.

A total of 52 studies were included through a multi-step selection process based on relevance to failure to rescue (FTR) after pancreatic surgery. The selection process is summarized in Figure 1, which follows the PRISMA 2020 format and includes the reason for exclusion.

We included 52 original studies published between 2010 and 2025. By design, these comprised retrospective analyses of national registries (RN, *n* = 24), retrospective single-center studies (RS, *n* = 13), retrospective multicenter studies (RM, *n* = 8), retrospective international multicenter studies (RI, *n* = 3), prospective national registry studies (PN, *n* = 2), prospective multicenter domestic studies (PM, *n* = 1), and prospective international multicenter studies (PI, *n* = 1). The number of institutions ranged from 1 (single center) to approximately 4000 (nationwide registry), and sample sizes ranged from 43 to 94,661 patients across the included studies. Review articles were excluded at the eligibility assessment and used only as background references. Study-specific details (authors, year, design, duration, number of institutions, and number of patients) are provided in Table 1.

Pancreatoduodenectomy (PD) was the most frequently reported procedure; distal pancreatectomy (DP) and total pancreatectomy (TP) were also commonly represented. All included cohorts comprised patients undergoing pancreatic surgery. Operative approach (pancreatic cohorts): reported in 22/52 studies; among the 20 with quantifiable shares (patient-weighted *n* = 121,632), final approach pooled to Open ≈91% and MIS ≈9% (LP ≈4%, Rb ≈0.5%); conversions counted under Open.

Although our search period spanned from 1992 onwards, no eligible studies specifically focusing on FTR in pancreatic surgery were identified prior to 2010. A single potentially relevant study by Ghaferi et al., published in 2009, was excluded due to the inclusion of multiple surgical procedures (AAA, esophageal surgery, CABG) beyond pancreatic surgery [80] and thus did not meet our inclusion criteria. Consequently, all included studies were published from 2010 onward.

### 3.2. Summary of FTR Descriptions

Of 52 eligible studies, 37 reported some form of failure-to-rescue (FTR). Three studies explicitly reported FTR by procedure and were split accordingly in Table 2 [67,74,77]. For the remaining 15 studies, FTR could not be explicitly determined because the definition and/or rate of FTR was not clearly reported; therefore, we include Table S1, which specifies the missing data for each study. 

#### 3.2.1. FTR Definition

The concept of FTR was originally proposed by Silber et al. in 1992, referring to the proportion of patients who died after experiencing major postoperative complications [19]. The principal dimensions were the observation time window, the severity threshold, the definition of complications, and the choice of denominator. FTR definitions clustered around H-CD3 (in-hospital, Clavien–Dindo [CD] ≥ III) and 90-CD3 (90-day cases, CD ≥ III), with additional use of 90-CD3a, 90-Acc3, and administrative/NSQIP variants. Across the entire dataset (40 entries, corresponding to 37 studies), the frequency of definitions by group was: G1 (90-day × CD ≥ III/IIIa/Acc3) in 6 studies, G2 in none, G3 (in-hospital/30-day × CD ≥ III/IIIa) in 9, G4 (90-day × Any, administrative) in 1, G5 (30-day/in-hospital × Any, non-CD) in 2, G6 (in-hospital × Any, administrative/NSQIP) in 9, G7 (90-day × specific complications such as CR-PPH/POPF) in 1, and G8 (alternative or non-comparable definitions) in 12.

#### 3.2.2. Time Windows, Severity Thresholds, and Denominators

All counts refer to 40 study entries. The distribution of observation windows was as follows: in-hospital only in 18 of 40 studies (45.0%), 90-day in 11 studies (27.5%), 30-day in 6 studies (15.0%), mixed in-hospital/30-day in 3 studies (7.5%), in-hospital plus 90-day in 1 study (2.5%), and composite windows in 1 study (2.5%). The severity thresholds applied were as follows: Clavien–Dindo-based definitions (CD ≥ III/IIIa or CD IV) in 20 of 40 studies (50.0%), Accordion ≥3 in 2 studies (5.0%), NSQIP serious/major criteria in 3 studies (7.5%), administrative or claims-defined “major” complications in 6 studies (15.0%), “any event” definitions in 8 studies (20.0%), and ISGPS-specific complications only in 1 study (2.5%). The denominators used were as follows: major complications defined by Clavien–Dindo or Accordion criteria in 20 of 40 studies (50.0%; CD-based in 18 and Accordion-based in 2), major complications identified through claims, ICD, or MSQC coding in 6 studies (15.0%), NSQIP serious/major morbidity in 3 studies (7.5%), any complication in 8 studies (20.0%), and narrow single-study denominators—mixed CD ≥ III or POPF, CD IV only, or ISGPS-specific complications only—in 1 study each (2.5%).

#### 3.2.3. Reported FTR Outcomes

Because no formal meta-analysis or risk-of-bias assessment was undertaken, the pooled values and between-group contrasts below are illustrative only. They should be interpreted as descriptive signals of how definitional choices affect apparent FTR, not as comparative effect estimates. All 95% confidence intervals are binomial (Wilson) around the simple pooled counts (Σn/ΣN) and do not incorporate between-study heterogeneity or study-level weighting.

Among studies using a 90-day window with CD ≥ III complications (G1), the pooled FTR was 13.2% (591/4461; 95% CI 12.3–14.3), with a study-level median 14.7% [10.9–19.5] (range 2.0–21.9%). Restricting capture to in-hospital (±30-day) outcomes at a similar CD threshold (G3) produced a pooled FTR of 10.3% (433/4213; 95% CI 9.4–11.2), with a median of 8.9% [7.1–13.4] (5.1–14.3%). Definitions centered on 30-day “serious/major” morbidity (G8) yielded the lowest pooled rate, 7.4% (1580/21,397; 95% CI 7.0–7.7), with a median of 9.6% [6.5–15.4] (5.3–33.3%). Descriptively, G1 exceeded G3 by 3.0 percentage points (RR 1.29) and G8 by 5.9 points (RR 1.79), while G3 exceeded G8 by 2.9 points (RR 1.39). These contrasts are descriptive only; no hypothesis testing was performed.

Groups that relied on administrative or registry “any/major” coding (G6) produced a pooled FTR of 11.5% (9353/81,332; 95% CI 11.3–11.7). The corresponding study-level median 7.8% [6.2–9.5] (2.9–12.6%) sat appreciably lower than the pooled rate, reflecting the disproportionate influence of very large cohorts on Σn/ΣN. Descriptively, G6 lay between G1 and G8: G1 vs. G6 differed by 1.7 points (RR 1.15), and G6 vs. G8 differed by 4.1 points (RR 1.56). No hypothesis testing was performed.

For complication-specific denominators (G7), exemplified by CR-PPH B/C and CR-POPF B/C, the pooled FTR for CR-PPH was 13.8% (9/65; 95% CI 7.5–24.3), and the combined study-level median across CR-PPH and CR-POPF was 7.6% [4.4–10.7]. Because the denominator is restricted to patients with a particular ISGPS event, these values are not directly comparable with the all-complication denominators used in G1, G3, and G8; they are best interpreted as event-specific fatality among patients who already crossed a high-risk threshold. Where only medians were available, 90-day “any” definitions (G4) clustered in the mid-teens (16.4% [14.9–17.9]; *n* = 2), in line with a broader denominator and extended follow-up. By contrast, 30-day/in-hospital “any” definitions (G5) centered lower (5.0% [4.1–7.0]; *n* = 4), echoing the attenuation observed whenever capture is truncated at discharge or 30 days.

We evaluated 15 studies that reported complications and/or mortality but could not be standardized to our FTR definition (90-day mortality among patients with CD ≥ III complications) (Table S1). Frequencies of not reported (NR) codes were: R6 (Counts missing): 8/15 (53.3%), R1 (Severity classification not reported): 7/15 (46.7%), R2 (Time window unspecified): 7/15 (46.7%), R4 (Denominator unclear): 5/15 (33.3%), R3 (No post-discharge death capture): 4/15 (26.7%), R8 (Other): 3/15 (20.0%), R5 (FTR concept not used): 2/15 (13.3%), and R7 (Mixed surgical case-mix): 1/15 (6.7%). Compound deficits were the rule, not the exception. The median number of NR reasons per study was 2 (mean 2.47); 13/15 (86.7%) studies had ≥2 codes.

### 3.3. Strategies for Reducing FTR in Pancreatic Surgery

We identified five major categories of strategies for reducing failure to rescue (FTR) in pancreatic surgery: (i) organizational or institutional interventions, (ii) evolution and safe implementation of surgical techniques, (iii) perioperative management, (iv) patient-related factors, and (v) non-technical skills (NTS). These categories were derived from the 52 included studies and are summarized in Table 3.

#### 3.3.1. Organizational Strategies

Centralization of pancreatic surgery to high-volume centers is widely recognized as a key strategy to reduce Failure to Rescue (FTR), particularly for high-risk procedures such as pancreaticoduodenectomy and total pancreatectomy. Across national and multi-center datasets, patients treated at high-volume hospitals consistently had lower failure-to-rescue (FTR) rates than those at low-volume hospitals, with absolute gaps of roughly 6–7 percentage points—for example, 12.0% vs. 6.4% [37], 11.1% vs. 5.4% [40], and 21.8% vs. 14.9% [38]—reinforcing a centralization signal beyond differences in complication rates alone. This is largely attributed to the presence of experienced multidisciplinary teams—including anesthesiologists, surgical staff, ICU specialists, interventional radiologists, emergency physicians, as well as a high-performing blood bank—that can promptly manage severe complications such as postoperative hemorrhage, pancreatic fistula, and septic shock [18,23,60,65,66,72,81]. These institutions are also characterized by the routine use of standardized clinical protocols for postoperative monitoring, reoperation criteria, antibiotic therapy, and ICU admission, ensuring timely and consistent responses across the care continuum [41,58,59,68]. Furthermore, continuous quality improvement is embedded in institutional culture through regular morbidity and mortality conferences, benchmarking initiatives, and structured case reviews, which collectively enhance team performance and clinical decision-making [38,63,67,70]. While the degree of centralization varies across countries depending on healthcare systems and geographic factors, the overall benefits of this model in improving FTR outcomes are consistently supported in both national and international studies [8,37,62,76,78].

#### 3.3.2. Evolution of Surgical Techniques

Advancements in surgical techniques, particularly the introduction and expansion of minimally invasive and robotic approaches, have significantly reshaped the landscape of pancreatic surgery [82]. These innovations offer several clinical advantages, including enhanced visualization, improved instrument dexterity, and more precise dissection, which are especially beneficial in complex procedures such as pancreaticoduodenectomy. Evidence suggests that these techniques can reduce intraoperative blood loss and postoperative complication rates, thereby potentially lowering the risks of Failure to Rescue (FTR) [54,77,83]. Consistent with these mechanistic expectations, comparative outcome studies report lower FTR after minimally invasive approaches than open surgery in selected populations—for example, a 90-day Medicare analysis found 13.4% with MIS versus 19.4% with open [42]. Procedure-specific contrasts also show systematically higher FTR after pancreatoduodenectomy (PD) than distal pancreatectomy (DP) across settings and definitions: 21.9% vs. 10.0% at 90 days [74]; 10.7% vs. 8.1% in standard NSQIP and 6.8% vs. 5.6% in pancreas-targeted NSQIP [65]; with longitudinal series preserving the PD > DP gradient despite overall improvements (PD 13.4→10.8→7.4% vs. DP 8.8→7.1→5.9% [63]. Additional cohorts echo this pattern—PD 20.5% versus total pancreatectomy 15.8% [77] and PD 7.5% versus DP 3.1% under CD ≥ IIIa denominators [67]. However, the adoption of advanced surgical techniques is accompanied by substantial technical complexity and steep learning curves with much variation by users. Early implementation of these technologies—especially in low-volume centers—has been associated with increased perioperative mortality, underscoring the need for deliberate institutional preparation, structured training, and careful case selection. To mitigate these risks, the introduction of minimally invasive pancreatic surgery should ideally occur within high-volume centers that possess the infrastructure to support safe implementation [25,64,69]. Several studies have emphasized the importance of structured training programs and stepwise adoption protocols in facilitating safe integration of novel techniques [63,69]. In this context, comprehensive institutional strategies—including readiness assessment to adopt the new technology, regular adverse event and near miss reporting, regular mortality and morbidity conferences [84] and root cause analyses, formal training curricula, mentorship by experienced surgeons, simulation training, and progressive case accumulation—have been shown to reduce technical errors and enhance patient safety [53,69,85]. Importantly, these frameworks enable community and non-academic hospitals to safely adopt complex procedures while maintaining acceptable outcomes [69].

In addition, specific technical strategies such as reducing intraoperative blood loss, optimizing anastomotic techniques, and minimizing the use of unnecessary prophylactics have been linked to improved outcomes [60,66,72]. For instance, experienced surgeons have adopted refined techniques such as pancreatojejunostomy over pancreaticogastrostomy, omitted the use of octreotide, and minimized intraoperative blood loss to improve outcomes in high-risk cases [60]. Similarly, center-level standardization of duct-to-mucosa anastomosis and tailored gland-specific strategies have shown a reduction in postoperative pancreatic fistula and mortality [72]. Furthermore, intraoperative hemorrhage and transfusion volume have been identified as independent predictors of FTR, reinforcing the importance of refined surgical technique and close anesthesiology collaboration to mitigate against excessive bleeding [60,86].

The focus of innovation in surgical technique has therefore shifted from mere technical feasibility to the development of safe, team-based, and context-appropriate implementation strategies. Meaningfully reducing FTR, requiresthe evolution of surgical techniques andalso how these techniques are embedded within systems of training, clinical governance, deploying a culture of deep team learning, and multidisciplinary collaboration [53,70,84].

#### 3.3.3. Improvements in Perioperative Management

Optimizing perioperative management is a critical component to reducing Failure to Rescue (FTR) after pancreatic surgery. Evidence supports a multifaceted approach combining clinical vigilance, structured protocols, and multidisciplinary coordination [23,26,53]. Timely recognition and intervention remain central. The adoption of early warning systems (EWS), real-time monitoring tools, and structured daily briefings can enhance situational awareness and prompt escalation of care [41,47,50]. Simulation-based training may also help frontline staff recognize subtle signs of deterioration and respond effectively [26,43]. Enhanced recovery protocols (ERAS)—including fluid management, early mobilization, and multimodal analgesia—have been shown to reduce postoperative complications and promote physiological resilience [39,56,70,87]. These system-based learning elements support earlier recovery and help prevent the progression of complications into life-threatening events. Daily multidisciplinary rounds involving surgeons, anesthesiologists, nurses, critical care physicians, and allied health professionals ensure alignment of care plans and facilitate proactive responses to complications [25,53]. Moreover, establishing explicit escalation pathways—with predefined thresholds and responsible role clarity—can enable rapid coordination during clinical deterioration [5,47].

Institutional implementation of standardized algorithms, morbidity and mortality (M&M) conferences, and benchmark tracking further strengthens accountability and shared learning [36,63,81]. When embedded into standard clinical practice, these strategies collectively foster a safety-oriented perioperative culture that mitigates the risks of FTR [20,23,50]. Several studies affirm these principles. Timely reoperation in deteriorating patients has been shown to be potentially lifesaving [50]. Improved rescue rates have also been associated with increased postoperative vigilance, early diagnostic imaging, and the preferential use of interventional radiology over reoperation in the management of complications [20,47,81]. Relevant process measures—such as time to diagnostic imaging or interventional radiology and documentation of escalation plans—have been proposed as practical quality indicators to evaluate the quality of the perioperative rescue.

#### 3.3.4. Consideration of Patient-Related Factors

Individual patient factors—such as frailty, nutritional status, comorbidities, and psychological resilience—profoundly influence postoperative outcomes and the likelihood of successful rescue in the event of complications [4,51,56,60]. Proactive risk stratification and personalized optimization planned and co-designed with patients and their caregivers are therefore essential components of any effective strategy to reduce FTR. Preoperative assessment tools, including prehabilitation programs as part of enhanced recovery after surgery (ERAS), should be employed to evaluate frailty, cardiopulmonary function, sarcopenia, and nutritional deficiencies [88]. Tailored interventions—such as exercise training to address functional deficits, nutritional supplementation, psychological support, and coaching toward healthier behaviors—can improve surgical readiness and physiological resilience [43,70,72]. Shared decision-making is particularly critical for high-risk patients. Involving patients and their families in discussions of operative risk, expected recovery, and potential complications not only supports informed consent but also sets realistic expectations and promotes engagement in perioperative care [25,58]. Multidisciplinary teams must develop individualized perioperative plans based on comprehensive risk profiles. Coordination with patients, informed by input from surgeons, anesthesiologists, nutritionists, physiotherapists, and psychosocial staff, ensures holistic management before and after surgery. These teams should also establish contingency strategies for high-risk individuals, including early access to intensive care and interventional radiology [18,43]., Aligning perioperative care with patient-specific vulnerabilities enhances resilience, facilitates timely rescue, and improves outcomes in pancreatic surgery [89]. As surgical candidates become increasingly complex, these personalized strategies become ever more vital to delivering safe and effective care [43,57,72].

#### 3.3.5. Emphasis on Non-Technical Skills (NTSs)

A closer synthesis of the reviewed literature reveals frequent references to elements such as communication, coordination, escalation of care, and teamwork. These appear primarily within broader discussions of institutional functioning and complication management. Although the term Non-Technical Skills (NTSs) was not used in any of the studies included in this systematic review, many of the descriptions align closely with what is recognized in the patient safety literature as NTS [90]. It is likely that the authors were not consciously framing their observations within the established NTS taxonomy.

### 3.4. Role of Non-Technical Skills (NTSs) in Reducing FTR

This section organizes the relevant findings according to the seven-domain framework proposed by Flin et al.: decision-making, situational awareness, communication, teamwork, leadership, managing stress, and coping with fatigue [91]. For each domain, we highlight how the reviewed literature—while not explicitly framed as NTS—offers insights into behaviors, structures, and practices that support timely rescue and safe perioperative care. Notably, among these domains, stress and fatigue management were not addressed in any of the 52 included studies, representing a critical gap in the current evidence.

An overview of the seven NTS categories, their subcomponents, and representative examples in the context of pancreatic surgery is summarized in Table 4.

#### 3.4.1. Decision-Making

Effective decision-making plays a central role in facilitating timely and appropriate responses to postoperative deterioration [92]. Several studies identified that failures in rescue were not primarily due to recognition or communication delays, but rather to delayed clinical action in response to deterioration, often stemming from insufficient escalation mechanisms [25,44,61]. Timeliness in decision-making can be supported by structured tools and protocols. Institutionalized clinical pathways such as “sepsis bundles” and time-outs have been suggested to help standardize responses to deterioration [37]. Predefined escalation protocols and clear systems of senior support are also considered essential to empower junior staff to act promptly when complications arise [25,44]. In the preoperative phase, aligning surgical strategies with individual risk profiles and patient preferences through shared decision-making is another approach to optimize outcomes [59]. Taken together, these insights indicate that improving decision-making capacity depends not only on individual clinical judgment, but also on embedding structured institutional strategies and decision-support tools throughout the surgical care continuum [93].

#### 3.4.2. Situational Awareness

Timely detection of postoperative deterioration is a critical prerequisite for effective rescue. Evidence suggests that failures to rescue are often influenced by the adequacy of situational awareness, including the ability to recognize early signs of physiological decline and initiate appropriate responses without delay. Structured monitoring systems such as Early Warning Scores (EWS) and continuous physiologic surveillance supported by electronic health records have been introduced to support early recognition of deterioration, particularly in patients who initially appear clinically stable [61]. Conditions such as postoperative shock, renal failure, and unplanned intubation have been identified as key triggers requiring immediate vigilance and diagnostic escalation [41,47]. Distributed monitoring responsibilities, especially among nursing staff, play a substantial role in enhancing situational awareness. More favorable nurse staffing (e.g., lower patient-to-nurse ratios) and attentive bedside observation have been associated with earlier detection of complications and more timely interventions [23,50]. Institutional readiness—including real-time access to radiology, intensive care, and experienced clinical personnel—also contributes to effective recognition and response. Facilities with well-coordinated multidisciplinary teams and clearly defined escalation pathways appear better equipped to detect and act upon early signs of severe postoperative complications [18,44,52,53,60,65].

Together, these findings suggest that situational awareness in the postoperative setting depends on the presense of a culture of patient safety supported by a combination of technological infrastructure, organizational design, and consistent clinical vigilance [94].

#### 3.4.3. Communication

Only a limited number of studies (*n* = 8) have explicitly addressed communication as a contributing factor in failure to rescue. Reported elements include escalation frameworks requiring direct notification of senior physicians [25,44], structured communication interventions such as technology-based systems [25], improved patient handovers using templated communication and interdisciplinary communication during critical moments [23,47,60]. Interestingly, one study reported that communication itself was not the limiting factor, as patient deterioration was recognized and conveyed but not acted upon promptly [61]. Additional studies addressed institutional communication strategies such as sharing outcomes transparently [63] and preoperative counseling [59].

Although the evidence is limited, these studies suggest that communication—across the institution and among clinical service members and especially between trainees—can influence rescue capacity through multiple pathways [95].

#### 3.4.4. Teamwork

Effective teamwork requires the integration of multidisciplinary expertise throughout the preoperative, intraoperative, and postoperative phases. Structured collaboration involving anesthesiologists, interventional radiologists, endoscopists, intensivists, and infection control professionals contributes to improved detection and management of complications, potentially reducing FTR [18,53,65]. Formal multidisciplinary team (MDT) meetings have been implemented at institutional and regional levels to enhance patient selection and care coordination across the surgical and cancer patient journey [63,78]. In settings with limited case volume, videoconference-based MDTs have been used to access external expert guidance and facilitate consistent perioperative standards. These practices reflect a broader shift toward team-based clinical governance and shared accountability in complex surgical patient management.

#### 3.4.5. Leadership

Leadership in complex surgical settings supports timely decision-making and effective team coordination. Among the 52 studies reviewed, only nine provided direct or indirect references to leadership-related factors, indicating a relative underrepresentation of this domain within the FTR literature. Identified barriers—such as hierarchical rigidity, lack of escalation protocols, and insufficient senior support—were found to impair complication response, underscoring the need for structured and responsive leadership that facilitates timely action and empowers clinical teams [25,44]. Furthermore, institutional strategies such as structured mentoring and expert supervision, particularly in the implementation of advanced procedures like robotic pancreaticoduodenectomy, reflect leadership investment in surgical safety and capacity-building [66,69].

#### 3.4.6. Stress and Fatigue

Stress and fatigue are known to degrade decision-making and undermine effective surgical teams. However, among the seven domains of non-technical skills (NTS) defined by Flin et al. [91], no study included in this review explicitly addressed stress management or fatigue management in the context of failure to rescue (FTR) after pancreatic surgery. Across the 52 reviewed studies, there were no references to interventions, assessments, or institutional strategies directly related to psychological stress, provider workload, fatigue, or duty-hour limitations as factors associated with rescue failure. This absence represents a notable gap in the literature.

## 4. Discussion

### 4.1. Statement of Main Findings

Three key messages emerge in reviewing the lead 52 studies on failure to rescue (FTR) after pancreatic surgery. First, reported FTR rates are not directly comparable because definitions vary widely by the observation window (in-hospital vs. 30 vs. 90-day), severity threshold (e.g., Clavien–Dindo ≥III/Iiia vs. any-complication denominators), and data sources—choices that alter both numerators and denominators and bias cross-study comparisons. Second, several structural and technical levers—regional centralization to high-volume centers, safe adoption and refinement of operative technique, and structured perioperative pathways with explicit escalation—are consistently associated with lower FTR. Third, the largest prospective opportunity to further reduce FTR lies in strengthening non-technical skills (NTS) in surgical teams—decision-making, situational awareness, communication, teamwork, and leadership—across the perioperative continuum. Given substantial heterogeneity and our descriptive synthesis (no formal meta-analysis), these conclusions are contextual and hypothesis-generating; see Section 5 for methodological limitations.

### 4.2. Strengthening Non-Technical Skills (NTS) to Reduce FTR: The Central Insight

#### 4.2.1. Why NTS Matters for Rescue

The most salient insight emerging from this narrative review is that strengthening non-technical skills (NTS) and enhancing medical team performance constitute a critical yet underdeveloped opportunity and a key strategy for reducing failure to rescue (FTR) after pancreatic surgery. While structural and technical domains such as centralization, surgical technique, and perioperative protocols have received substantial attention, timely recognition and coordinated response to deterioration are often compromised by breakdowns in decision-making, communication, and leadership. FTR is not solely a function of complication severity or surgical complexity—it frequently results from cognitive, behavioral, and team-based failures that delay or obstruct rescue. This reframes FTR mitigation as not only a matter of infrastructure but also of behavioral and organizational cultural dynamics within clinical teams. In the context of medical safety, insufficient team coordination, ambiguous leadership, and reluctance to speak up have been identified as frequent contributors to adverse outcomes, even in technically advanced environments [96]. These issues reflect deeply rooted deficits in present medical training in NTS, which are rarely addressed through standard quality improvement initiatives [97,98].

Failure to rescue a patient often unfolds during moments of clinical uncertainty—subtle physiological deviations, ambiguous signs, or shifting care priorities—when rapid interpretation of evolving risks, role clarity, coordinated interdisciplinary action, and confident escalation are required. In such situations, it is not technical expertise but vigilance, situational awareness, collaborative sense-making, and assertive decision-making that prove decisive. While the importance of non-technical skills (NTS) has been widely recognized in the intraoperative setting, with structured systems such as the Non-Technical Skills for Surgeons (NOTSS) formalizing decision-making, communication, and leadership within the operating theater [99], similar capacities are equally critical in the postoperative phase. Rescue failure often occurs not because a complication is undetectable, but because teams fail to recognize or respond to it on time.

Conceptual frameworks describing team-based vigilance, shared mental models, and adaptive expertise provide compelling justification for extending NTS principles beyond the operating room [100]. However, structured frameworks alone are insufficient. Their successful application depends on the environment in which they are enacted. In actual clinical settings, the execution of NTS is often hindered by latent organizational human factors that impair team functioning [101]. Rigid hierarchies, ambiguous role expectations, and suppressed speaking-up behaviors—common in high-stakes medical environments—can prevent even well-trained teams from taking timely and appropriate action [102,103]. These barriers are not merely operational; they are deeply rooted in medical clinical disciplines and institutional cultures. Foundational work in safety science, notably by Leape (1994), Reason (1997), and Vaughn (1997), has described how normalization of deviance and diffusion of responsibility can allow latent hazards to persist undetected until they culminate in harm [104,105,106]. These dynamics are particularly dangerous when time is critical, as they impair the team’s ability to make sense of the impending FTR and mount an effective rescue to transform manageable complications into preventable deaths.

#### 4.2.2. Evidence Gap in the Current Literature

Despite widespread recognition of the importance of non-technical skills (NTS) in ensuring patient safety, their integration into surgical outcomes research—especially in the context of failure to rescue (FTR)—remains markedly limited. In this systematic review A few studies in this systematic review explicitly examin how cognitive and behavioral processes such as decision-making, escalation of care, situational awareness, or communication influenced the progression from time of surgical complication to mortality. This omission reveals a critical blind spot in the current literature, which continues to emphasize structural factors over behavioral dynamics. Most FTR studies still focus on institutional metrics such as hospital volume, ICU staffing, or surgical expertise, while largely neglecting how interprofessional teams function and learn in real time under duress during episodes of patient deterioration [80]. Even when adverse events are reported, the root causes related to human performance—such as delayed recognition, lack of role clarity, communication breakdowns, or poor team coordination—are rarely acknowledged or analyzed in a systematic fashion. Frameworks developed to assess NTS, such as the Non-Technical Skills for Surgeons (NOTSS) taxonomy [99], have largely been restricted to intraoperative or simulation environments, with minimal application to the postoperative settings where most FTR events arise. Similarly, observant clinical studies of real-world team behaviors in clinical care have revealed that latent threats and communication failures often go undetected in routine practice, yet these findings remain poorly integrated into the FTR research. In addition, foundational safety science work by Vincent et al. (2004) has emphasized that outcomes are shaped not only by systems and protocols but also by shared mental models, coordination, and adaptability—core components of NTS [100]. The lack of behavioral monitoring and team-based evaluation in the postoperative phase thus represents a missed opportunity to intervene before failure occurs. Bridging this evidence gap requires intentional study designs that can capture real-time team interactions, cognitive load, and communication flow during the critical window between complication and rescue and translate these domains into measurable and auditable quality indicators. Only by incorporating these human factors can future research move toward a more comprehensive and actionable model of perioperative surgical safety. Notably, none of the reviewed studies addressed stress or fatigue management, despite their established relevance to cognitive performance, clinical vigilance, and decision-making under pressure [107]. These omissions highlight a critical blind spot in the behavioral dimensions of rescue that remains unexplored in current surgical literature.

### 4.3. Current Landscape and Definition-Driven Limitations of FTR

#### 4.3.1. Definition Heterogeneity and Interpretability

As detailed in the Results section (see Table 2), estimates of failure to rescue (FTR) vary systematically with the observation window (in-hospital vs. 30 vs. 90 days), the severity threshold (e.g., CD ≥ III/Iiia vs. broader “any-complication” denominators), and the data source (clinical registries, NSQIP, administrative claims). The directional signal is consistent across definition groups (G1–G8): wider time windows and higher severity thresholds yield higher—and clinically tighter—FTR estimates. These definitional choices are not neutral; they embed ascertainment biases that can alter cross-center or cross-country comparisons and may even invert comparative statements if definitions differ. Because the FTR is a ratio *among patients with complications*, broad denominators (e.g., “any complication”) dilute the ratio, whereas restricting to intervention-requiring events (CD ≥ III/IIIa) tightens attribution to genuine “rescue” opportunities [23,37,47,75]. NSQIP commonly anchors 30-day follow-up; administrative claims often lack validated severity mapping; and registry designs differ in post-discharge capture. For interpretability and comparability, definition elements must be declared up front and held constant in primary analyses.

#### 4.3.2. Studies Not Reporting FTR and Why It Matters

Missing a severity threshold (R1) prevents anchoring the denominator to CD ≥ III; an unspecified time window (R2) makes the numerator scope (in-hospital vs. 30 vs. 90-day mortality) non-comparable; and absent counts (R6) preclude calculating *n*/N even when a percentage is provided. Because our standardized FTR relies on 90-day deaths among CD ≥ III, any one of these deficits can invalidate re-tabulation; in this subset, they were present in every study (often jointly). When the complication cohort is undefined or mixed (R4), “rescue” is diluted or inflated depending on whether minor or organ-specific endpoints are included. In-hospital-only follow-up (R3) systematically underestimates FTR by missing post-discharge deaths—precisely where delayed deterioration and the need for effective rescue are most apparent. A minority of studies either did not use the FTR construct (R5) or mixed PD/DP/TP without stratification (R7). These are structural rather than reporting problems: even with perfect counts, such designs would remain ill-suited for comparative FTR benchmarking.

Examples include specialty-restricted denominators (e.g., POPF grade B/C only) or time windows that could not be uniquely identified from the text/tables. These choices make the reported “FTR-like” numbers non-mappable to 90 d × CD ≥ III.

#### 4.3.3. The Persistent Blind Spot: Post-Discharge Rescue

Many studies rely on in-hospital ascertainment, omitting deaths that occur soon after discharge. This decision point disproportionately skews evaluation of team-based detection and escalation, because recognition, early re-intervention, and safe readmission pathways operate close to the discharge boundary. Routine incorporation of post-discharge status via national registries, cross-system EHR linkages, or prospective 90-day follow-up is essential [42,52,74,108].

#### 4.3.4. A Reference Definition: 90-Day FTR Among CD ≥ III Complications

The Clavien–Dindo classification provides an internationally adopted, behaviorally meaningful threshold at grade III/IIIa, i.e., complications that mandate procedural or operative intervention—the precise domain in which “rescue” is actionable [109]. This aligns naturally with contemporary pancreas-specific taxonomies: the ISGPS 2016 update re-grounded POPF as clinically relevant (CR-POPF B/C), explicitly tying definition to therapeutic consequence and resource mobilization, conceptually congruent with CD ≥ III [110]. Where legacy datasets use alternative scales, the Accordion severity *grading* system offers a defensible cross-walk to CD, enabling harmonization without distorting the rescue construct [111].

Pancreatectomy mortality accrues materially after 30 days; restricting ascertainment to in-hospital or 30-day endpoints systematically misses deaths that are tightly coupled to postoperative complications and their rescue pathways. Evidence from oncology pancreatectomy shows that 90-day mortality substantially exceeds 30-day mortality, underscoring the clinical relevance of a longer window [112]. More broadly, health-services analyses demonstrate that including post-discharge deaths changes hospital performance signals, reinforcing that shorter windows underestimate failure to rescue impact [108]. Contemporary international practice is converging on this standard: a recent 67-country prospective snapshot of pancreatic surgery proposed and operationalized modern outcome definitions with extended follow-up, illustrating feasibility and global face validity [49].

FTR is not merely “mortality per complication”; it sits within goals-of-care decisions and cultural norms around escalation [113]. Given these definition-driven constraints, our synthesis is descriptive and hypothesis-generating; see Section 5 for methodological limitations.

### 4.4. Practical Implementation Strategies for Embedding NTS in Surgical Practice

Strengthening NTS requires translating conceptual frameworks into measurable and auditable process indicators:

(a) Decision-making

Implementation of structured tools such as early warning scores, SBAR communication protocols, escalation checklists, and predefined contingency plans reduces ambiguity and inertia during deteriorating situations. These cognitive aids support timely escalation and mitigate delay-related FTR [105,114]. Simulation-based training has also been shown to improve decision fluency in complex scenarios [115]. 

Indicators: EWS compliance rates, SBAR utilization, time from abnormality to escalation, senior review documentation, simulation participation and performance.

(b) Situational awareness tools

Maintaining shared situational awareness is essential for early recognition of patient deterioration. Team huddles, risk dashboards, and cross-disciplinary briefings can enhance team alignment and vigilance across shifts and roles [97]. These mechanisms prevent fragmented monitoring and promote anticipatory care.

Indicators: Huddle implementation rates, frequency of nursing observations, dashboard review adherence, and time from warning sign to escalation.

(c) Structured communication frameworks

Miscommunication remains one of the leading contributors to preventable harm in surgery. Standardized handover protocols, such as SBAR, and the adoption of closed-loop communication practices reduce misunderstanding and information loss during crises [116,117,118]. These approaches enhance clarity, especially under stress.

Indicators: Handover completeness, closed-loop adherence, and documentation of calls escalated to senior staff.

(d) Team training and simulation

High-fidelity simulation and scenario-based team training build collective reflexes, support shared mental models, and foster environments where team members can speak up regardless of hierarchy [115]. TeamSTEPPS, developed by the Agency for Healthcare Research and Quality (AHRQ), provides evidence-based tools and strategies to enhance team performance and patient safety [119]. Its implementation in clinical settings has demonstrated improvements in patient outcomes, underpinned by improved communication, leadership behaviors, and mutual support across diverse surgical environments [120].

Indicators: Frequency of MDT meetings, staff participation rates, simulation training uptake, and TeamSTEPPS implementation audits.

(e) Leadership and role of clarity

Clear definition of escalation roles, responsibility handoffs, and leadership expectations during critical phases improves coordination and response speed [95].

Ambiguity in team structure and leadership has been shown to contribute to confusion, duplication, and diffusion of responsibility [97,105]. In the context of anesthesia and critical care, poor role delineation was associated with delayed or inappropriate actions during crises [121]. Regular reinforcement of shared accountability norms is essential to overcome these latent system failures.

Despite their relatively low cost and strong theoretical foundation, NTS strategies are rarely implemented in a structured, system-wide manner within surgical practice. To reduce preventable mortality after complex procedures, NTS must be treated not as soft skills but as critical, trainable, and measurable competencies embedded in clinical routines, protocols, and institutional culture. 

Indicators: Presence of written escalation protocols, chain-of-command documentation, and leadership training participation rates.

(f) Stress and fatigue

Individual and team stress levels and fatigue levels domains are critical to cognitive performance although absent from the reviewed studies.

Indicators: Monitoring duty hours, compliance with rest breaks, stress evaluation tool completion, and institutional fatigue management systems.

### 4.5. Future Directions for Research and Implementation

Future research and implementation efforts must address several critical gaps to effectively translate non-technical skills (NTS) into measurable improvements in surgical outcomes, especially in the high-risk context of pancreatic cancer surgery. First, the field must prioritize the establishment of standardized and universally accepted definitions of FTR, including consistent thresholds for timing, severity, and complication types. The current heterogeneity across studies limits comparability and precludes effective benchmarking. Consensus definitions would enable robust cross-institutional and cross-national research, including in low- and middle-income settings where surgical infrastructure and rescue capabilities are limited and vary widely in quality. Second, prospective multicenter studies and international collaborative registries using standardized definitions are urgently needed. These initiatives should capture not only structural variables and outcomes but also process- and organizational context-level data on team behavior, escalation practices, and communication flow [122]. Given the complexity and high complication burden of pancreatic procedures, the creation of a global registry focused on FTR after pancreatic surgery could provide critical insights into modifiable contributors to mortality. Third, targeted investigations into the specific, quantifiable impact of non-technical skills on FTR reduction are warranted. Rather than reiterating individual process indicators, future studies should focus on validating surgery-specific NTS measures and determining their association with rescue outcomes. Fourth, the development and validation of standardized NTS metrics is essential to enable benchmarking across healthcare institutions and countries. These measures should be piloted and refined in high-risk units such as hepato-biliary-pancreatic surgery services. Fifth, simulation-based research can serve as a powerful modality for testing team interventions in safe, controlled environments. High-fidelity simulations of common postoperative complications—such as delayed hemorrhage, anastomotic leak, or intra-abdominal sepsis—can be used to evaluate escalation reflexes, speaking-up behavior, and leadership role clarity under pressure. These simulations can also inform training program design and system-level improvements. Sixth, dedicated applications of implementation science methodologies and tools are necessary to ensure that NTS-based strategies are sustainably integrated into real-world surgical practice. Barriers such as hierarchical culture, unclear role expectations, and resistance to change must be identified and addressed. In pancreatic surgery, where care is delivered across multiple disciplines and settings, successful implementation requires institution-wide coordination and cultural adaptation. Seventh, surgical training and accreditation frameworks must evolve to include formal NTS training not only in the operating room but throughout the perioperative continuum. In addition to technical mastery, trainees must develop fluency in recognizing clinical deterioration, initiating timely escalation, team engagement and support, and learning to lead coordinated team-based responses. These competencies should be assessed early and longitudinally and treated as core qualifications for independent surgical practice. Finally, emerging technologies, including continuous physiological monitoring, machine learning algorithms, and predictive analytics, have potential for enhancing early detection and guiding rescue efforts [123]. However, their deployment must be rigorously evaluated through prospective trials to determine their safety, effectiveness, integration feasibility, and cost–benefit balance—particularly in the complex environment of postoperative pancreatic care. Ultimately, reducing FTR in pancreatic surgery requires a paradigm shift, from viewing mortality as an inevitable outcome of technical complexity to understanding it as a preventable system’s failure of recognition, team coordination, and effectively mounted responses.

## 5. Limitations

This systematic review with narrative synthesis has several limitations that merit acknowledgment. First, although we followed a PRISMA-based search and selection [35], we did not preregister a protocol (e.g., PROSPERO) [124] and we did not perform a formal risk-of-bias assessment (e.g., ROBINS-I for nonrandomized studies [125], RoB 2 for randomized trials [126]). In conjunction with the substantial heterogeneity in FTR definitions, outcomes, and study designs across the included studies, these omissions limit the certainty of our findings and constrain their generalizability. Nonetheless, the use of predefined inclusion/exclusion criteria, independent dual screening, and consensus-based data extraction helped mitigate these risks. Second, our literature search was limited to the PubMed database, which may have omitted relevant studies indexed elsewhere or in the gray literature, introducing the possibility of publication bias [127]. Third, restricting the search to English-language publications may have introduced language bias by excluding relevant studies published in other languages [128]. Fourth, the included studies exhibited substantial heterogeneity in terms of FTR definitions (time frames and severity thresholds), limiting comparability across studies. Accordingly, we did not conduct a formal meta-analysis; while we report a small number of exploratory pooled estimates generated in Excel, these are descriptive only, and our conclusions primarily reflect qualitative (narrative) synthesis [129]. We therefore could not quantify between-study inconsistency or examine small-study effects, further constraining the precision and generalizability of the results [21,130]. Fifth, most included studies were conducted in high-income countries and large academic centers, limiting generalizability to settings with different healthcare resources and infrastructures [131]. Sixth, the majority of the included studies used retrospective, observational designs, thereby limiting data accuracy, raising concerns about unmeasured confounding, and restricting causal inference [132]. In particular, selection and information biases (e.g., inconsistent case capture and variable FTR definitions) could have inflated apparent benefits of centralization or specific techniques, while residual data confounding at the hospital level may partially account for observed between-center differences in FTR. Seventh, although non-technical skills (NTS) have emerged as critical to reducing FTR, the variability in their measurement methods and definitions across studies prevents objective quantification and synthesis of their effectiveness; moreover, core NTS domains such as stress and fatigue were seldom measured, precluding any quantitative assessment of their impact, although the success of NTS implementation and team training in other surgical domains demonstrates their potential [133,134]. Finally, we did not assess the certainty of evidence using formal approaches such as GRADE [135], which further limits the strength of inference. Taken together, the absence of a formal quality appraisal and our decision not to undertake a formal meta-analysis because of study heterogeneity are limitations that affect the strength and generalizability of our conclusions; accordingly, our findings should be interpreted as contextual and hypothesis-generating rather than definitive.

## 6. Conclusions

Failure to rescue (FTR) remains a critical vulnerability in pancreatic surgery, reflecting persistent challenges in the timely recognition and coordinated management of major postoperative complications. We observed across 52 studies, substantial heterogeneity in how FTR is defined and measured and, consequently, wide variation in reported rates. Convergent signals nevertheless point to actionable safety opportunities: regional centralization to high-volume centers; safe adoption and refinement of surgical technique; structured perioperative management with explicit escalation pathways; patient-specific risk stratification and prehabilitation; and deliberate integration of non-technical skills (decision-making, situational awareness, communication, teamwork, and leadership) among surgical teams and across the care continuum.

We strongly recommend to enable fair benchmarking and reduce reporting variability, a pancreas-specific operational definition of FTR—numerator: all-cause 90-day mortality; denominator: patients with major complications (Clavien–Dindo ≥ III/IIIa). Given the heterogeneity across studies and the absence of a formal quality assessment and meta-analysis in our review, our synthesis should be interpreted as descriptive. To make this definition actionable and auditable, centers should report a concise set of standardized, process-level rescue indicators that capture the timeliness of recognition, escalation, and definitive treatments. Examples include time to diagnostic imaging, time to interventional radiology or reoperation, and adherence to predefined escalation protocols.

Embedding new measurements and training of non-technical skills within routine quality programs—and evaluating these strategies across diverse resource settings and referral networks—should be prioritized. Building reliable patient rescue systems in which deterioration is detected early, escalation is unequivocal, and definitive treatment is delivered without delay offers the most credible path to lowering FTR and improving survival after pancreatic surgery.

## Figures and Tables

**Table 1:** Summary of key failure to rescue (FTR) studies: Authors, year, design, study period, number of institutions, number of patients, data source, and procedure mix.

Authors	Year	Design	Duration	No. of Institutions	No. of Patients	Data Source	Procedure Mix (PD/DP/TP) & Approach
Ghaferi et al. [23]	2010	RN	2000–2006	672	8862	NIS (HCUP-AHRQ) + AHA Annual Survey	NR (identified by pancreatectomy ICD-9-CM codes; PD/DP/TP breakdown not reported)
Haigh et al. [36]	2011	RN	2005–2007	183	2610	ACS NSQIP participant-use files	PD 100% (classic or pylorus-preserving)
Amini et al. [37]	2015	RN	2000–2011	1802	35,986	HCUP Nationwide Inpatient Sample	PD 51.7%; DP 33.3%; TP 5.6% (Others 9.4%) (Open 94.9%; MIS 5.1%)
Healy et al. [38]	2015	RM	2008–2013	19	1007	Michigan Surgical Quality Collaborative (MSQC)	PD 62.1% (Whipple 48.1% + PP-Whipple 14.0%); DP 34.1%; TP 1.8% (Open 100%)
Tamirisa et al. [25]	2016	RM	2011–2012	37	2694	ACS NSQIP Pancreatectomy Demonstration Project	PD 64.4%; DP 30.7%; TP 3.0%
Carr et al. [39]	2017	RS	2013–2015	1	254	ACS NSQIP (QITI) + institutional PD database	PD 100%
Gani et al. [40]	2017	RN	2002–2011	~1834	11,081	HCUP Nationwide Inpatient Sample (AHRQ)	PD 65.8%; DP 24.6%; TP 3.7% (Open 95%; MIS 5.0%)
Varley et al. [41]	2017	RN	2005–2012	NR	4514	ACS NSQIP PUF	PD 100%
Capretti et al. [26]	2018	RM	2010–2013	7	856	Prospectively collected hospital databases, centrally merged at Humanitas	PD 61.8%; DP 28.2%; TP 10.0% (LP 10.7%)
Chen et al. [42]	2018	RN	2013–2015	NR	15,140	MEDPAR Inpatient Files + Denominator File	PD/DP/TP breakdown NR (Open 90.6%; MIS 9.4%)
El Amrani et al. [4]	2018	RN	2012–2015	NR (exact count not specified)	12,333	PMSI (ICD-10 + French procedure classification; linked administrative data)	PD 68.9%; DP 26.9%; TP 3.2%; central 1.0%
Krautz et al. [8]	2018	RN	2009–2014	~654	60,858	Nationwide DRG statistics (Federal Statistical Office & Länder RDC)	PD (proximal) 60.3%; DP 26.5%; TP 8.0% (remaining: segmental 2.8%; other partial 2.4%)
Pecorelli et al. [43]	2018	RM	2008–2015	3	120	Institutional prospective databases at three university-affiliated high-volume centers; preoperative CT assessed centrally	PD 100%; DP 0%; TP 0%
van Rijssen et al. [44]	2018	PN	2014–2015	18	1342	Dutch Pancreatic Cancer Audit (DPCA)	PD 100%
Cerullo et al. [45]	2019	RN	2010–2014	NR	3280	Truven Health Analytics MarketScan Commercial Claims & Encounters	PD 93.3%; TP 6.7%
Diaz et al. [46]	2019	RM	2005–2016	189	23,014	California OSHPD hospital discharge database	PD 100%
Gleeson et al. [47]	2019	RN	2005–2012	NR	5027	ACS NSQIP PUF	PD 100%; DP 0%; TP 0%
Merath et al. [48]	2019	RN	2013–2015	737	13,873	MEDPAR Inpatient Files linked with Denominator File; AHA Survey; Medicare cost reports (wage index)	NR
Sánchez-Velázquez et al. [5]	2019	RI	2012–2015	23	2375	Prospective databases from each center centrally pooled (whipplebenchmarks.org)	PD 100% (Open 100%)
van Roessel et al. [49]	2019	RS	1992–2017	1	1434	Institutional prospective database; survival from National Cancer Registry	PD 100% (PPPD 81.9%; Whipple 18.1%) (Open 96.4%; LP 3.6%)
Wroński et al. [50]	2019	RS	2003–2017	1	43	Single institution (Medical University of Warsaw)	PD 100%; DP 0%
Bhatti et al. [51]	2020	RS	2011–2018	1	116	Single-institution hospital records	PD 100% (standard PD 70%; PPPD 19%; PD + organ resection 11%; vascular resection 12%)
El Amrani et al. [18]	2020	RN	2012–2018	NR (nationwide; all hospitals)	19,938	PMSI (ICD-10 diagnoses + CCAM procedures)	PD 75.0%; DP 23.9%; CP 0.7%; TP 0.4%
Nymo et al. [52]	2020	RN	2015–2016	5	394	NoRGast national quality registry + EPJ cross-check; deaths via National Registry linkage	PD 100%
Endo et al. [53]	2021	RN	2011–2014	≈4000	422	National Clinical Database (NCD)	HPD (which includes PD as a component) 100%; Major HPD (60%); Minor HPD (40%)
Gleeson et al. [20]	2021	RI	2014–2017	≈224	22,983	ACS NSQIP; DPCA; SNPPCR; DGAV StuDoQ Pancreas	PD 100% (MIS 6.0%)
Lequeu et al. [54]	2021	RN	2009–2018	631	10,632	PMSI (Programme de Médicalisation des Systèmes d’Information)	DP 100% (Open 77.0%; LP 23.0%)
Pastrana et al. [55]	2021	RN	2006–2016	~121–680	32,165	ACS NSQIP PUF	PD 100%
Bassi et al. [56]	2022	RS	2000–2019	1	2989	Institutional database (prospectively collected; retrospectively analyzed)	PD 100% (PPPD 86.0%; Whipple 14.0%)
Di Gioia et al. [57]	2022	RS	2010–2019	1	1865	University of Verona Hospital Trust (Pancreas Institute) prospective database; retrospectively analyzed	PD 100%
Sutton et al. [58]	2022	RS	2013–2020	1	637	Institutional NSQIP (100% capture of pancreatectomies); MPOG (intra-op); hospital cost administrative data (USD)	Whipple/Total pancreatectomy 63%; DP/RAMPS 37% (Open 81.5%; MIS 18.5% by back-calculatin)
van Beek et al. [59]	2022	RM	2008–2019	2	123	Hospital surgical databases & histopathology archives; electronic patient records	PD/PPPD 41.5%; DP 40.7%; TP 4.9% (enucleation 9.8%; combined 3.3%) (Open 66.7%; LP 13.0%; Rb 20.3%)
Fukada et al. [60]	2023	RS	2010–2022	1	177	Single-institution hospital dataset (JSHBPS-certified training institution)	NR (mixed HPB procedures; PD/DP/TP distribution unclear)
Li et al. [61]	2023	RS	2011–2020	1	58	Single-center HPB prospective registry + retrospective chart completion	PD 100%
Moazzam et al. [62]	2023	RN	2013–2017	677	19,625	100% Medicare Standard Analytic Files (SAFs)	NR
Suurmeijer et al. [63]	2023	PN	2014–2019	18 → 16	5345	Dutch Pancreatic Cancer Audit (DPCA; DPCG)	PD 79%; DP 21%
Theijse et al. [64]	2023	RN	2014–2021	NR (all national DPCG centers)	1402	Dutch Pancreatic Cancer Audit (DPCA)	PD 100% (PPPD 48.2%; PRPD 12.6%; Classic Whipple 39.2%) (Open 74.6%; Rb 20.3%; LP 5.11%)
Vawter et al. [65]	2023	RN	2014–2019	NR	45,157	ACS NSQIP standard & pancreas-targeted registries	PD 67%; DP 33%
Cannas et al. [66]	2024	RI	2003–2023	18	8189	Pancreas Fistula Study Group dataset	PD 100% (MIS 3.8%)
de Graaff et al. [67]	2024	RN	2014–2021	24	7365	DHBA, DPCA (managed by DICA)	PD 78.9%; DP 21.1% (Open 68.8%; LP 26.8%; 4.4%, minor variable-level missingness remains)
Duclos et al. [68]	2024	RM	2014–2018	21	1188	21 high-volume centers (data collected at each site)	DP 100% (Open 52.8%; LP 41.4%; Rb 5.8%)
Heckman et al. [69]	2024	RS	2016–2022	1	65	Prospectively maintained institutional database + ACS NSQIP	PD 100% (Rb 100%)
Henry et al. [70]	2024	RN	2014–2019	17	149	Dutch Pancreatic Cancer Audit (DPCA); PORSCH	PD 82%; DP 11%; TP 7% (Open 88%; LP 7%; Rb 6%)
Khalid et al. [71]	2024	RM	2014–2023	NR (multicenter health-system EHR registry)	314	Northwell Health multicenter pancreatic cancer database (EHR abstracted to REDCap)	PD (classical; PPPD; LP; Rb;); DP (open; LP; Rb); TP
Kinny-Köster et al. [72]	2024	RS	2003–2021	1	156	Prospective institutional pancreatectomy registry	PD 100% (Open 96.8%; Rb 3.2%)
Leech et al. [73]	2024	RS	1999–2023	1	79	Pancreatic resection registry (UCT/Groote Schuur)	PD 100% (PPPD 98.7%; Classical 1.3%)
PancreasGroup.org Collaborative [74]	2024	PI	2021	354	4223	PancreasGroup.org electronic CRF (mandatory outcome fields)	PD 59.3%; DP 24.5%; TP 6.9%; Enucleation 1.5%; Other 5.2% (percentages may not sum to 100%) (Open 83.9%; LP 11.6%; Rb 4.5%)
Patel et al. [75]	2024	RN	2014–2020	NR (multicenter NSQIP)	15,790	ACS NSQIP Pancreatectomy Targeted	PD/DP/TP (PD/DP/TP distribution unclear)
Wang et al. [76]	2024	RS	2015–2022	1	995	Single hospital database (The First Hospital of Jilin University)	PD 100% (LP 100%)
Capretti et al. [77]	2025	PM	2016–2022	5	277	Prospectively collected data from participating centers	PD 72.9%; TP 27.1%
Tschaidse et al. [78]	2023	RN	2014–2019	~60	3011	StuDoQ|Pancreas (DGAV) registry	PD 80.1%; DP 19.9% (Open 94%; LP 5.7%)
Uttinger et al. [79]	2025	RN	2010–2020	939	94,661	German DRG billing data (Federal Statistical Office)	PD 61.2%; DP 26.6%; TP 9.9%

PD: pancreatoduodenectomy (Whipple); PP: pylorus-preserving; PRPD: pylorus-resecting PD; DP: distal pancreatectomy; TP: total pancreatectomy; CP: central pancreatectomy. LP: laparoscopic; Rb: robotic; MIS: minimally invasive surgery; RAMPS: radical antegrade modular pancreatosplenectomy; HPD: hepatopancreatoduodenectomy. NR: not reported; “–” = not applicable/excluded. ACS NSQIP, DPCA, DPCG, PMSI, MEDPAR, NoRGast, DGAV, DICA, OSHPD, AHRQ follow their standard registry/agency names.

**Table 2:** The literature explicitly defining and reporting failure to rescue (FTR) metrics.

Authors	Year	Definition	Time Window	Severity Threshold	Denominator	Reported FTR (*n*/N, %)	Post-Discharge Capture
Capretti et al. (PD) [77]	2025	90-CD3 [G1]	90 days	CD ≥ III (≥IIIa)	CD ≥ III complication	15/73 (20.5%)	Yes (90-day follow-up; method NR)
Capretti et al. (TP) [77]	2025	90-CD3 [G1]	90 days	CD ≥ III (≥IIIa)	CD ≥ III complication	3/19 (15.8%)	Yes (90-day follow-up; method NR)
Lequeu et al. [54]	2021	90-CD3 [G1]	90 days	CD ≥ III	CD ≥ III complication	355/3153 (11.2%)	Unclear (national in/outpatient linkage, external death ascertainment NR)
Nymo et al. [52]	2020	90-Acc3 [G1]	90 days	Accordion ≥ 3 (re-anchored to CD ≥ III)	Major complication (Accordion ≥ 3)	17/125 (13.6%)	Yes (national registry linkage incl. cross-regional EPJ)
PancreasGroup.org Collaborative (PD) [74]	2024	90-CD3a [G1]	90 days	CD ≥ IIIa	Major-complication (CD ≥ IIIa)	157/717 (21.9%)	Yes (prospective 90-day follow-up via CRF; ascertainment method NR)
PancreasGroup.org Collaborative (DP) [74]	2024	90-CD3a [G1]	90 days	CD ≥ IIIa	Major-complication (CD ≥ IIIa)	20/203 (10.0%)	Yes (prospective 90-day follow-up via CRF; ascertainment method NR)
Pecorelli et al. [43]	2018	90-CD3 [G1]	90 days	CD ≥ III (III–IV)	CD ≥ III complication	23/120 (19.2%)	Yes (90-day mortality; method NR)
van Beek et al. [59]	2022	90-CD3 [G1]	90 days	CD ≥ III	CD ≥ III complication	1/51 (2.0%)	Unclear (90-day mortality adopted; ascertainment NR)
Bassi et al. [56]	2022	H-CD3 [G3]	In-hospital	CD ≥ III	Major complications (CD ≥ III)	70/597 (11.7%)	No (in-hospital only)
de Graaff et al. (PD) [67]	2024	H/30-CD3a [G3]	In-hospital/30 days	CD ≥ IIIa	Major complications (CD ≥ IIIa)	7.5% (1.6–28.5%)	No (in-hospital/30 d)
de Graaff et al. (DP) [67]	2024	H/30-CD3a [G3]	In-hospital/30 days	CD ≥ IIIa	Major complications (CD ≥ IIIa)	3.1% (0–14.9%)	No (in-hospital/30 d)
Di Gioia et al. [57]	2022	H-CD3 [G3]	In-hospital	CD ≥ III	Major complications (CD ≥ III)	57/404 (14.1%)	No (in-hospital only)
Fukada et al. [60]	2023	H-CD3 [G3]	In-hospital	CD ≥ III	Major complications (CD ≥ III)	9/177 (5.1%)	No (in-hospital only)
Leech et al. [73]	2024	H-CD3 [G3]	In-hospital	CD ≥ III	Major complications (CD ≥ III)	3/21 (14.3%)	No (in-hospital only)
Suurmeijer et al. [63]	2023	H-CD3 [G3]	In-hospital	CD ≥ III	Major complications (CD ≥ III)	PD 54/404 > 46/426 > 34/462 (13.4 > 10.8 > 7.4%); DP 5/57 > 6/84 > 5/85 (8.8 > 7.1 > 5.9%)	No (in-hospital/30 d; 90 d not obtained)
Theijse et al. [64]	2023	H-CD3a [G3]	In-hospital	CD ≥ IIIa	Major complications (CD ≥ IIIa)	57/642 (8.9%)	No (DPCA −30 d only)
van Rijssen et al. [44]	2018	H-CD3 [G3]	In-hospital	CD ≥ III	Major complications (CD ≥ III)	56/391 (14.3%)	No (in-hospital only)
van Roessel et al. [49]	2019	H-CD3 [G3]	In-hospital	CD ≥ III	Major complications (CD ≥ III)	31/463 (6.7%)	No (in-hospital only)
Gleeson et al. [20]	2021	H-CD3-Mix [G3]	In-hospital	CD ≥ III or ISGPS POPF B/C (mixed)	CD ≥ III or POPF B/C (mixed)	8.20%	No (in-hospital only)
Chen et al. [42]	2018	90-Any-Admin [G4]	90 days	Any (administrative)	Any complication	Pancreas Open 19.4%, MIS 13.4%	Yes (Medicare denominator file)
Carr et al. [39]	2017	H/30-Any [G5]	30 d/In-hospital	Any (non-CD)	Any complication	Fellow 6%, Resident 4%	Unclear (30 d and in-hospital mixed)
Haigh et al. [36]	2011	30-Any-NSQIP [G5]	30 days	NSQIP “≥1 morbidity”	“≥1 morbidity”	older 10.1% vs. younger 4.1%	Yes (NSQIP 30 d)
Amini et al. [37]	2015	H-Any-Admin [G6]	In-hospital	Other (ICD-9 “major”)	Any complication (ICD-9 “major”)	All 9.0%; LV 12.0%; IV 8.5%; HV 6.4%	No (in-hospital only; NIS)
Cerullo et al. [45]	2019	H-Any-Admin [G6]	In-hospital	Other (claims “major”)	Major complications (claims)	27/920 (2.9%)	No (in-hospital only)
El Amrani et al. [18]	2020	H-Any-Admin [G6]	In-hospital	Other (administrative “major”)	≥1 major complications	940/10,758 (8.7%)	No (in-hospital only)
Gani et al. [40]	2017	H-Any-Admin [G6]	In-hospital	Any (AHRQ)	Any complication	LV 11.1%, IV 7.1%, HV5.4%	No (in-hospital only)
Ghaferi et al. [23]	2010	H-Any-Admin [G6]	In-hospital	Other (ICD-9 “major”)	Major complications (ICD-9)	Quintile of hospital mortality (6.4–40.0)	No (in-hospital only)
Tamirisa et al. [25]	2016	H-Any-NSQIP [G6]	In-hospital	Any (NSQIP events)	Any complication	34/1111 (3.1%)	No (in-hospital only)
Uttinger et al. [79]	2025	H-Any-Admin [G6]	In-hospital	Any (administrative/registry)	Any complication	8040/64,029 (12.6%)	No (in-hospital only)
Varley et al. [41]	2017	H-Any-NSQIP [G6]	In-hospital	Any (NSQIP major/minor)	Any complication	312/4514 (6.9%)	No (in-hospital only)
Duclos et al. [68]	2024	90-Spec [G7]	90 days	ISGPS CR-PPH B/C and/or CR-POPF B/C	Specific complications (CR-PPH *n* = 65; CR-POPF *n* = 202)	CR-PPH 9/65 (13.8%); CR-POPF 1.3% (*n* NR)	Yes (90-day outcome capture; method NR)
Cannas et al. [66]	2024	90-Acc3-Alt [G8]	90 days	Accordion ≥3	Severe complications (Accordion ≥ 3)	182/1533 (11.9%)	Yes (90-day follow-up)
Gleeson et al. [47]	2019	30-NSser-NSQIP [G8]	30 days	NSQIP serious/major	Serious/major morbidity	361/5027 (7.2%)	Yes (NSQIP 30 d)
Healy et al. [38]	2015	30-Alt [G8]	30 days	MSQC major (non-Clavien)	Major complications (MSQC)	LV 21.8%, HV 14.9%	Yes (registry 30 d)
Li et al. [61]	2023	H + 90-CD4 [G8]	In-hospital + 90 days	CD IV	CD IV patients	19/58 (33.0%)	Unclear (90 d capture; method NR)
Pastrana Del Valle et al. [55]	2021	30-NSmaj-NSQIP [G8]	30 days	NSQIP major morbidity	Major morbidity	Yearly % 9.8→4.1 (2006→2016)	Yes (NSQIP 30 d)
Patel et al. [75]	2024	30-CD3-NSQIP [G8]	30 days	CD ≥ III (NSQIP mapped)	Major complications (CD ≥ III)	245/4623 (5.3%)	Yes (NSQIP 30 d)
Vawter et al. [65]	2023	30-NSser-NSQIP [G8]	30 days	NSQIP serious morbidity	Serious morbidity	Standard NSQIP: PD 184/1720 (10.7%); DP 47/578 (8.1%); Pancreas-targeted NSQIP: PD 400/5871 (6.8%); DP 94/1681 (5.6%)	Yes (NSQIP 30 d)
Wang et al. [76]	2024	H-Any-Alt [G8]	In-hospital	Other (enumerated “major”)	“Major complications” (denominator NR)	24 (2.4%)	No (in-hospital only)
Endo et al. [53]	2021	Comp-Any [G8]	Composite (in-hospital ≤90 days + 30 days post-discharge)	Any (non-CD)	Any complication	33.3%/17.0%/9.3% (22/66; 8/47; 18/193)	Partial (composite window)

Definitions: G1: FTR90—Clavien–Dindo grade ≥III, G2: FTR30—Clavien–Dindo grade ≥III, G3: In-hospital—Clavien–Dindo grade ≥III, G4: FTR90—Any complication, G5: FTR30—Any complication, G6: In-hospital—Any complication, G7: Specific complication (e.g., POPF grade B/C as the index event), G8: Alternative/Non-comparable definitions, Severity threshold: CD: Clavien–Dindo; ISGPS: International Study Group of Pancreatic Surgery; NSQIP: National Quality Improvement Project; ICD: International Classification of Diseases; Agency for Healthcare Research and Quality; MSQC: Michigan Surgical Quality Collaborative; Reported FTR (*n*/N, %): PD: pancreatoduodenectomy (Whipple); DP: distal pancreatectomy; MIS: minimally invasive surgery; LV: low volume; IV: intermediate volume; HV: high volume; CR: clinically relevant; PPH: postpancreatectomy hemorrhage; POPF: postoperative pancreatic fistula, Post-discharge capture: NR: not reported; EPJ: electronic patient journal; CRF: cade report form; DPCA: Dutch Pancreatic Cancer Audit; NIS: Nationwide Inpatient Sample.

**Table 3:** Summary of intervention strategies identified for reducing failure to rescue (FTR) in pancreatic surgery.

Authors	Organizational/Institutional Factors	Surgical Technique	Perioperative Management	Patient- Related Factors	Non-Technical Skills (NTS)
Ghaferi et al. [23]	✔		✔		✔
Haigh et al. [36]	✔	✔	✔	✔	✔
Amini et al. [37]	✔		✔		
Healy et al. [38]	✔	✔	✔		
Tamirisa et al. [25]	✔	✔	✔	✔	✔
Carr et al. [39]	✔	✔	✔		
Gani et al. [40]	✔		✔		
Varley et al. [41]	✔		✔	✔	✔
Capretti et al. [26]	✔		✔		✔
Chen et al. [42]		✔		✔	
El Amrani et al. [4]	✔		✔	✔	
Krautz et al. [8]	✔				
Pecorelli et al. [43]	✔			✔	
van Rijssen et al. [44]	✔		✔	✔	✔
Cerullo et al. [45]	✔		✔	✔	
Diaz et al. [46]	✔			✔	
Gleeson et al. [47]	✔		✔	✔	✔
Merath et al. [48]	✔				
Sánchez-Velázquez et al. [5]	✔				✔
van Roessel et al. [49]		✔	✔		
Wroński et al. [50]	✔	✔	✔		✔
Bhatti et al. [51]	✔	✔	✔		
El Amrani et al. [68]	✔		✔	✔	
Nymo et al. [52]	✔		✔		
Endo et al. [53]	✔	✔	✔		✔
Gleeson et al. [20]	✔		✔		
Lequeu et al. [54]	✔	✔		✔	
Pastrana et al. [55]	✔		✔		
Bassi et al. [56]	✔	✔	✔	✔	✔
Di Gioia et al. [57]	✔	✔	✔	✔	
Sutton et al. [58]	✔		✔	✔	✔
van Beek et al. [59]	✔	✔	✔	✔	✔
Fukada et al. [60]	✔	✔	✔		✔
Li et al. [61]	✔	✔	✔		
Moazzam et al. [62]	✔				
Suurmeijer et al. [63]	✔	✔	✔	✔	
Theijse et al. [64]	✔	✔	✔	✔	
Vawter et al. [65]	✔		✔		✔
Cannas et al. [66]	✔	✔	✔	✔	
de Graaff et al. [67]	✔		✔		
Duclos et al. [68]	✔	✔	✔		
Heckman et al. [69]	✔	✔		✔	
Henry et al. [70]	✔	✔	✔	✔	✔
Khalid et al. [71]	✔			✔	
Kinny-Köster et al. [72]	✔	✔	✔	✔	
Leech et al. [73]	✔	✔		✔	
Patel et al. [75]			✔		
Uttinger et al. [79]	✔		✔		
Wang et al. [76]				✔	
PancreasGroup.org Collaborative [74]	✔		✔		
Capretti et al. [77]	✔	✔	✔		
Tschaidse et al. [78]	✔	✔			

**Table 4:** Main categories and elements of non-technical skills.

Category	Elements
Situation awareness	Gathering information
	Interpreting information
	Anticipating future states
Decision-making	Defining the problem
	Considering options
	Selecting and implementing an option
	Outcome review
Communication	Sending information clearly and concisely
	Including context and intent during information exchange
	Receiving information, especially by listening
	Identifying and addressing barriers to communication
Team working	Supporting others
	Solving conflicts
	Exchanging information
	Coordinating activities
Leadership	Using authority
	Maintaining standards
	Planning and prioritizing
	Managing workload and resources
Managing stress	Identifying the symptoms of stress
	Recognizing the effects of stress
	Implementing coping strategies
Coping with fatigue	Identifying the symptoms of fatigue
	Recognizing the effects of fatigue
	Implementing coping strategies

## Data Availability

No new data were created or analyzed in this study. Data sharing is not applicable to this article.

## Supplementary Materials

The following supporting information can be downloaded at https://www.mdpi.com/article/10.3390/cancers17193259/s1. Table S1: Reasons studies could not be standardized to the reference FTR definition.

## Abbreviations

The following abbreviations are used in this manuscript:

PD	Pancreatoduodenectomy
POPF	Postoperative Pancreatic Fistula
PPH	Postpancreatectomy Hemorrhage
FTR	Failure to Rescue
PRISMA	Preferred Reporting Items for Systematic Reviews and Meta-Analyses
HBP	Hepato-Biliary-Pancreatic
NSQIP	National Surgical Quality Improvement Program
RN	Retrospective Analyses of National Registries
RS	Retrospective Single-Center Studies
RM	Retrospective Multicenter Studies
RI	Retrospective International Multicenter Studies
PN	Prospective National Registry Studies
PM	Prospective Multicenter Domestic Studies
PI	Prospective International Multicenter Studies
NIS	Nationwide Inpatient Sample
HCUP	Healthcare Cost and Utilization Project
AHRQ	Agency for Healthcare Research and Quality
AHA	American Hospital Association (Annual Survey)
PUF	Participant-Use File
MSQC	Michigan Surgical Quality Collaborative
MEDPAR	Medicare Provider Analysis and Review
PMSI	Programme de Médicalisation des Systèmes d’Information
DRG	Diagnosis-Related Group
RDC	Research Data Center
DPCA	Dutch Pancreatic Cancer Audit.
OSHPD	Office of Statewide Health Planning and Development
SNPPCR	Swedish National Pancreatic & Periampullary Cancer Registry
StuDoQ	StuDoQ|Pancreas registry
DGAV	German Society for General and Visceral Surgery
NCD	National Clinical Database
JSHBPS	Japanese Society of Hepato-Biliary-Pancreatic Surgery
MPOG	Multicenter Perioperative Outcomes Group
DICA	Dutch Institute for Clinical Auditing
DHBA	Dutch Hepato-Biliary Audit
PORSCH	Dutch Stepped-Wedge “Algorithm-Based Care” Program/Trial After PD
CRF	Case Report Form
EPJ	Electronic Patient Journal
ISGPS	International Study Group of Pancreatic Surgery
ICD	International Classification of Diseases
HV/IV/LV	High/Intermediate/Low Volume
PP	Pylorus-Preserving Pancreaticoduodenectomy
PRPD	Pylorus-Resecting Pancreaticoduodenectomy
DP	Distal Pancreatectomy
TP	Total Pancreatectomy
CP	Central Pancreatectomy
LP	Laparoscopic
Rb	Robotic
MIS	Minimally Invasive Surgery
RAMPS:	Radical Antegrade Modular Pancreatosple-Nectomy
AAA	Abdominal Aortic Aneurysm
CABG	Coronary Artery Bypass Grafting
HPD	hepatopancreatoduodenectomy
ICU	Intensive Care Unit
EWS	Early Warning Score/System
ERAS	Enhanced Recovery After Surgery
IR	Interventional Radiology
MDT	Multidisciplinary Team
EHR	Electronic Health Record
SBAR	Situation, Background, Assessment, Recommendation
NTS	Non-Technical Skills
NOTSS	Non-Technical Skills for Surgeons
PROSPERO	International Prospective Register of Systematic Reviews
ROBINS-I	Risk of Bias in Non-randomized Studies of Interventions
RoB 2	Risk of Bias 2
GRADE	Grading of Recommendations Assessment, Development and Evaluation
