# Supplementary material for: Failure to Rescue After Surgery for Pancreatic Cancer: A Systematic Review and Narrative Synthesis of Risk Factors and Safety Strategies
Source: Cancers (Basel). 2025 Oct 8;17(19):3259. doi: 10.3390/cancers17193259 (PMC12523307; doi:10.3390/cancers17193259)
Supplement: Supplementary file 1 [file cancers-17-03259-s001.zip › cancers-3840498-supplementary.pdf]

Table S1. Reasons studies could not be standardized to the reference FTR definition (90-day mortality among patients with Clavien–Dindo ≥ III complications).

Authors	Year	Missing Elements	NR Reason Codes
Capretti et al. [26]	2018	FTR n/N missing (only % reported); number of ≤90 days deaths within CD≥III unknown; denominator restricted to POPF B/C and incompatible	R6, R8
El Amrani et al. [4]	2018	No severity framework such as Clavien/ISGPS; N_CD≥III not reported; ≤90 days deaths within CD≥III unknown; post-discharge death capture unclear	R1
Krautz et al. [8]	2018	No mapping to Clavien; N_CD≥III unknown; ≤90 days deaths within CD≥III unknown; post-discharge deaths not captured	R1, R3
Diaz et al. [46]	2019	Severity framework unclear; FTR n/N missing; denominator definition unclear; no 90 days follow-up (in-hospital only)	R1, R4, R6, R3
Merath et al. [48]	2019	N_CD≥III and ≤90 days deaths within CD≥III not reported; severity not specified; mixed surgical procedures	R1, R7
Sánchez-Velázquez et al. [5]	2019	FTR time window unclear; n/N for 90 days FTR within CD≥III missing	R2, R6

Wroński et al. [50]	2019	N_CD≥III not reported; number of ≤90 days deaths within CD≥III unknown; time window (30/90 days) unclear; denominator for all complications not presented	R2, R8
Bhatti et al. [51	2020	Time window not specified; N_CD≥III not reported; number of ≤90 days deaths within CD≥III unknown	R2
Sutton et al. [58]	2022	No FTR definition; denominator unknown; severity unclear; N_CD≥III/≤90 days deaths not reported	R5, R1, R4
Moazzam et al. [62]	2023	Severity unclear; FTR time window unclear; CD≥III denominator and count of 90 days deaths missing; FTR n/N not presented	R1, R2, R6
Kinny-Köster et al. [72]	2024	Severity threshold unclear; denominator unknown; n/N not presented; in-hospital follow-up only (no 90 days capture); FTR rate itself not a comparative endpoint	R1, R3, R4, R5, R6
Heckman et al. [69]	2024	FTR time window unclear; denominator unknown; n/N missing; severity threshold not specified	R2, R4, R6
Henry et al. [70]	2024	FTR denominator unknown; FTR n/N missing; no post-discharge death capture; re-tabulation elements (N_CD≥III and 90 days deaths) missing	R4, R6, R3
Khalid et al. [71]	2024	FTR time window unclear; N_CD≥III not reported; ≤90 days deaths within CD≥III unknown; method for capturing post-discharge deaths unclear	R2, R6
Tschaidse et al. [78]	2023	Death time window unclear; method for capturing post-discharge deaths unclear; number of ≤90 days deaths within CD≥III unknown	R2, R8

NR: Not reported, R1: Se-verity classification not reported, R2: Time window unspecified, R3: No post-discharge death capture, R4: Denominator unclear, R5: FTR concept not used, R6: Counts missing , R7: Mixed surgical case, R8: Other
